# Supplementary material for: The First Genetic Linkage Map of Winged Bean [Psophocarpus tetragonolobus (L.) DC.] and QTL Mapping for Flower-, Pod-, and Seed-Related Traits
Source: Plants (Basel). 2022 Feb 12;11(4):500. doi: 10.3390/plants11040500 (PMC8878720; doi:10.3390/plants11040500)
Supplement: Supplementary file 1 [file plants-11-00500-s001.zip › Figure S1 A genetic linkage map of the winged bean [Psophocarpus tetragonolobus (L.) DC.] constructed from an 86 F2 population, derived from the W054 xTPT9 cros.pdf]

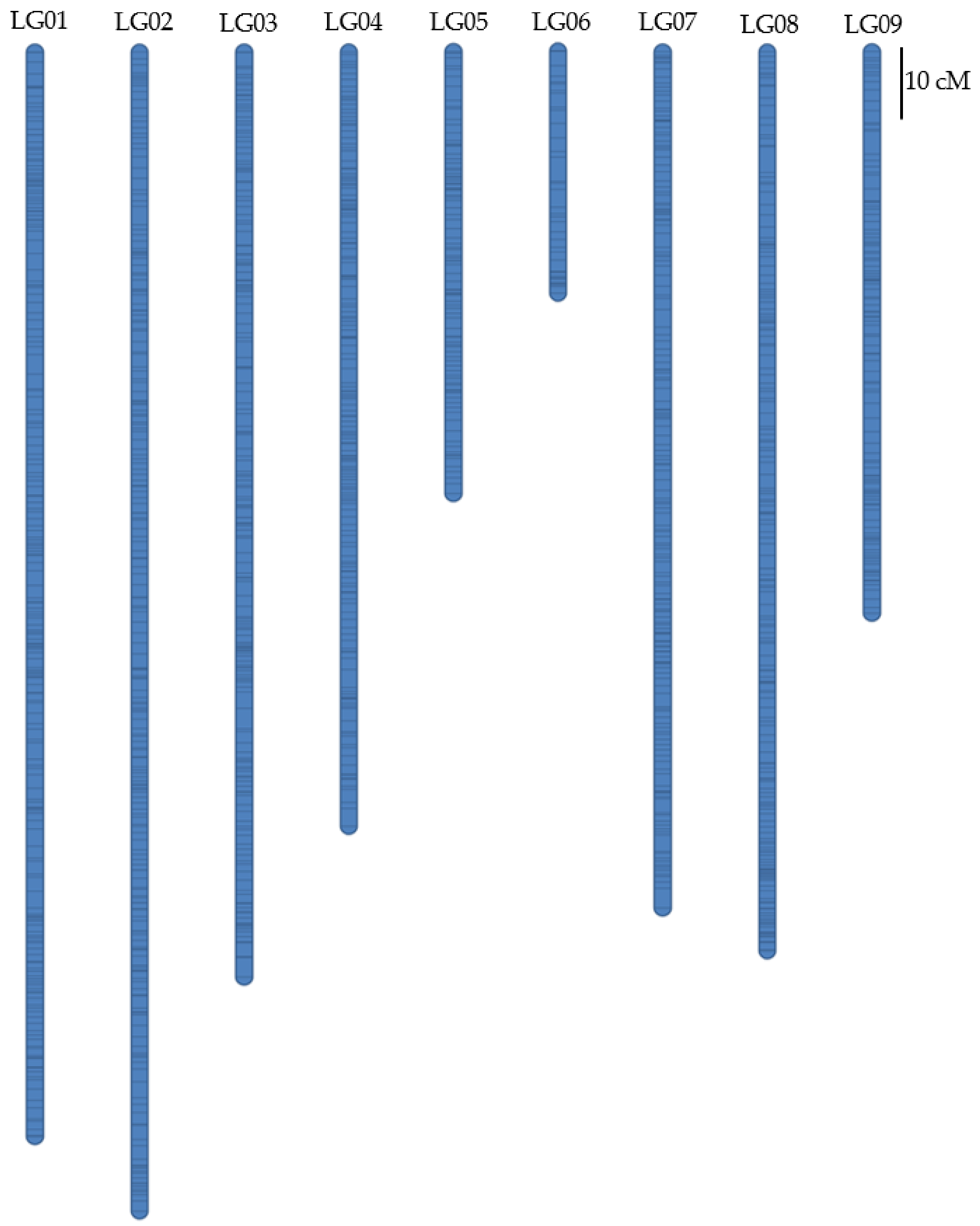

Figure S1. A genetic linkage map of the winged bean [*Psophocarpus tetragonolobus* (L.) DC.] constructed from an 86 F_2 population, derived from the W054 x TPT9 cross, using SNP markers.
